# Radiosensitizing effect of intratumoral interleukin-12 gene electrotransfer in murine sarcoma

**DOI:** 10.1186/1471-2407-13-38

**Published:** 2013-01-29

**Authors:** Ales Sedlar, Simona Kranjc, Tanja Dolinsek, Maja Cemazar, Andrej Coer, Gregor Sersa

**Affiliations:** 1 Department of Experimental Oncology, Institute of Oncology Ljubljana, Ljubljana, Slovenia; 2 Faculty of Health Sciences, University of Primorska, Izola, Slovenia

**Keywords:** IL-12, Gene electrotransfer, Irradiation, Sarcoma, Mice

## Abstract

**Background:**

Interleukin-12 (IL-12) based radiosensitization is an effective way of tumor treatment. Local cytokine production, without systemic shedding, might provide clinical benefit in radiation treatment of sarcomas. Therefore, the aim was to stimulate intratumoral IL-12 production by gene electrotransfer of plasmid coding for mouse IL-12 (mIL-12) into the tumors, in order to explore its radiosensitizing effect after single or multiple intratumoral gene electrotransfer.

**Methods:**

Solid SA-1 fibrosarcoma tumors, on the back of A/J mice, were treated intratumorally by mIL-12 gene electrotransfer and 24 h later irradiated with a single dose. Treatment effectiveness was measured by tumor growth delay and local tumor control assay (TCD_50_ assay). With respect to therapeutic index, skin reaction in the radiation field was scored. The tumor and serum concentrations of cytokines mIL-12 and mouse interferon γ (mIFNγ) were measured. Besides single, also multiple intratumoral mIL-12 gene electrotransfer before and after tumor irradiation was evaluated.

**Results:**

Single intratumoral mIL-12 gene electrotransfer resulted in increased intratumoral but not serum mIL-12 and mIFNγ concentrations, and had good antitumor (7.1% tumor cures) and radiosensitizing effect (21.4% tumor cures). Combined treatment resulted in the radiation dose-modifying factor of 2.16. Multiple mIL-12 gene electrotransfer had an even more pronounced antitumor (50% tumor cures) and radiosensitizing (86.7% tumor cures) effect.

**Conclusions:**

Single or multiple intratumoral mIL-12 gene electrotransfer resulted in increased intratumoral mIL-12 and mIFNγ cytokine level, and may provide an efficient treatment modality for soft tissue sarcoma as single or adjuvant therapy to tumor irradiation.

## Background

Novel therapeutic approaches, such as gene therapy, often complement established ones: surgery, chemotherapy or radiotherapy. Cytokines, such as tumor necrosis factor (TNF-α), interleukins (IL-2, IL-12) and interferons (IFN-α, IFN-β, IFN-γ), as recombinant molecules have already proved effective for radiosensitization in preclinical studies [1,2]. Their clinical use, however, has been hampered by toxic systemic peak concentrations during repetitive intravenous administration [1,3,4]. A new method of such tumor radiosensitization is gene therapy with plasmids coding for cytokines, the advantage being their sustained and controlled release [5].

One of the immunostimulatory cytokines with a good antitumor and radiosensitizing effect is IL-12 [6]. It exhibits antitumor effects that can be ascribed to the stimulation of an immune response with augmented natural killer cell and cytotoxic T cell activity and also to anti-angiogenic effects [7]. Possible mechanisms of IL-12 radiosensitization are enhanced tumor antigen presentation due to radiation induced apoptosis [8], anti-angiogenic effects [9], and the production of radiosensitizer nitric oxide [10]. The prevailing mechanism of radiosensitization, however, is not clear and it might be influenced by factors such as tumor type and the timing of the therapy. The radiosensitizing effect of IL-12 has already been reported for recombinant IL-12, as well as gene therapy with IL-12 performed with viral [8,9,11-16] and non-viral gene transfer [17-19]; in combination with tumor irradiation, a synergistic antitumor effect on local tumor growth and distant metastases has been demonstrated. Gene electrotransfer, utilizing the application of electric pulses to the tissue for naked plasmid DNA transfer into the cells [20-22] is a safe, non-toxic transfection method as efficient as viral vectors. IL-12 gene electrotransfer can be performed either intratumorally, with a predominantly local effect, or into the muscle, with a systemic effect on primary tumors and metastases [20,21,23,24]. The systemic effectiveness of intramuscular IL-12 gene electrotransfer, with a high tumor cure rate, has been demonstrated in subcutaneous murine LPB and SA-1 sarcomas, as well as in induced SA-1 lung metastases. In addition, in both tumor models, systemic release of IL-12 had a good radiosensitizing effect [17]. Sarcomas are a specific clinical situation in which tumor irradiation alone cannot provide suitable local control of larger, unresectable tumors [25-27]. Combining irradiation with intratumoral IL-12 gene electrotransfer in order to obtain a local radiosensitizing effect could thus be beneficial for the treatment of bulky sarcomas.

Our study therefore explores the potential radiosensitizing effectiveness of mIL-12 plasmid electrotransfer to sarcoma tumors. Tumor and serum concentrations of cytokines mIL-12 and mIFNγ were determined, as well as antitumor effectiveness, degree of tumor radiosensitization and skin reaction in the irradiation field. In addition to single, a radiosensitizing effect was also determined for multiple intratumoral mIL-12 gene electrotransfer in murine sarcoma.

## Methods

### Animals and tumors

Experiments were performed on A/J mice of both sexes, 12–14 weeks old and weighing 20–25 g. Mice were purchased from the Medical Experimental Centre, Institute of Pathology, Faculty of Medicine, University of Ljubljana (Slovenia) and were held at the Institute of Oncology Ljubljana in a specific-pathogen-free animal colony at controlled temperature and humidity, with a 12 h light/dark cycle. Solid SA-1 fibrosarcoma tumors (Jackson Laboratory, Bar Harbor, ME) were induced in the back of the syngeneic A/J mice, by subcutaneous injection of 5 × 10^5^ SA-1 cells [28]. When tumor volumes reached approximately 40–50 mm^3^, mice were subjected to a protocol specific to each therapeutic group (listed in Tables 1 and 2). The protocols were approved by the Ministry of Agriculture and Environment of the Republic of Slovenia (permission no. 34401-10/2009/6).

**Table 1:** Antitumor effectiveness of single mIL-12 gene electrotransfer alone or combined with irradiation on SA-1 sarcoma

**Therapeutic group**	**N**	DT (days; AM ± SE)*	GD (days; AM ± SE)**	**Cures**^**† **^**(%; n)**	**SC resistance**^**#**^
Control	12	1.7 ± 0.2	-	0	
EP	13	4.2 ± 0.6	2.5 ± 0.6	0	
dsRed	12	3.1 ± 0.3	1.3 ± 0.3	0	
EGT dsRed	14	5.3 ± 0.9	3.5 ± 0.9	0	
mIL-12	13	3.1 ± 0.4	1.4 ± 0.4	0	
EGT mIL12	14	20.0 ± 3.0 ^‡^	18.3 ± 3.0	7.1 (1/14)	0/1
IR	13	5.4 ± 0.9	3.7 ± 0.9	0	
EP + IR	14	14.4 ± 4.2 ^‡^	12.7 ± 4.2	0	
dsRed + IR	9	5.3 ± 1.1	3.6 ± 1.1	0	
EGT dsRed + IR	11	9.3 ± 1.9	7.5 ± 1.9	0	
mIL-12 + IR	13	10.7 ± 1.7	8.9 ± 1.7	0	
EGT mIL-12 + IR	14	32.6 ± 4.3 ^‡**§**^	30.9 ± 4.3 ^**§**^	21.4 (3/14)	1/3

Therapeutic groups: 10 Gy single dose irradiation (IR), electrical pulse application alone (EP) or combined with irradiation (EP + IR), intratumoral injection of plasmid DNA coding for mIL-12 or dsRed alone (mIL-12, dsRed) or combined with irradiation (mIL-12 + IR, dsRed + IR), mIL-12 or dsRed gene electrotransfer alone (EGT mIL-12, EGT dsRed) or combined with irradiation (EGT mIL-12 + IR, EGT dsRed + IR).

N - Number of all mice in the group.

* - Tumor doubling time - only mice with tumors were included in calculation.

** - Tumor growth delay - only mice with tumors were included in calculation.

^†^ - Cures were determined 100 days after the treatment.

^#^ - Resistance to secondary challenge – number of cured mice that were resistant to secondary challenge is shown.

^**‡**^

**-** Statistical significant difference compared to control group (p < 0.05).

^§^ - Statistical significant difference compared to IR (p < 0.05).

**Table 2:** Antitumor effectiveness of triple mIL-12 gene electrotransfer alone or combined with irradiation on SA-1 sarcoma

**Therapeutic group**	**N**	DT (days; AM ± SE)*	GD (days; AM ± SE)**	**Cures**^**† **^**(%; n)**	**SC resistance (n)**^**#**^
Control	10	3.4 ± 0.4	-	0	
3× EP	10	6.0 ± 1.0	2.7 ± 1.0	0	
3× dsRed	10	3.1 ± 0.3	−0.3 ± 0.3	0	
3× EGT dsRed	12	6.8 ± 0.8	3.4 ± 0.8	8.3 (1/12)	1/1
3× mIL-12	13	5.2 ± 1.3	1.9 ± 1.3	0	
3× EGT mIL-12	14	17.7 ± 5.4 ^**‡**^	14.3 ± 5.4	50.0 (7/14)	7/7
IR 10 Gy	10	8.1 ± 1.5	4.8 ± 1.5	0	
3× EP + IR	11	13.1 ± 1.9	9.7 ± 1.9	27.3 (3/11)	3/3
3× dsRed + IR	12	9.9 ± 1.8	6.5 ± 1.8	16.7 (2/12)	2/2
3× EGT dsRed + IR	12	25.7 ± 4.8 ^**‡§**^	22.3 ± 4.8	25.0 (3/12)	3/3
3× mIL-12 + IR	14	10.2 ± 1.7	6.9 ± 1.7	7.1 (1/14)	1/1
3× EGT mIL-12 + IR	15	43.1 ± 28.8 ^**‡§**^	39.8 ± 28.8 ^**‡**^	86.7 (13/15)	13/13

Therapeutic groups: 10 Gy single dose irradiation (IR), triple electric pulse application alone (3× EP) or combined with irradiation (3× EP + IR), triple intratumoral injection of plasmid DNA coding for mIL-12 or dsRed alone (3× mIL-12, 3x dsRed) or combined with irradiation (3× mIL-12 + IR, 3× dsRed + IR), triple mIL-12 or dsRed gene electrotransfer alone (3× EGT mIL-12, 3× EGT dsRed) or combined with irradiation (3× EGT mIL-12 + IR, 3× EGT dsRed + IR).

N - Number of all mice in the group.

* - Tumor doubling time - only mice with tumors were included in calculation.

^**^ - Tumor growth delay - only mice with tumors were included in calculation.

^†^ - Cures were determined 100 days after the treatment.

^#^ - Resistance to secondary challenge – number of cured mice that were resistant to secondary challenge is shown.

^‡^ - Statistical significant difference compared to control group (p < 0.05).

^§^ - Statistical significant difference compared to IR 10 Gy (p < 0.05)

### Plasmid DNA

Therapeutic plasmid encoding mIL-12 (pORF-mIL-12, InvivoGen, Toulouse, France) and control plasmid encoding red fluorescent protein (pORF-dsRed, constructed in our laboratory) were isolated using a Qiagen Maxi Endo-Free Kit (Qiagen, Hilden, Germany) and diluted to a concentration of 1 mg/ml.

### Gene electrotransfer

Plasmid DNA (20 μl) was injected intratumorally and 10 minutes later 2 sets of 4 electric pulses were applied to the tumor in perpendicular directions (600 V/cm, 5 ms, 1 Hz). Pulses were delivered with electric pulse generator GT-01 (Faculty of Electrical Engineering, Ljubljana, Slovenia) using stainless steel parallel plate electrodes. The distance between the electrodes was 6 mm and they were placed on the skin enclosing the tumor that was approximatelly 5–6 mm long, with approximate height of 2.5 mm. A thin layer of conductive gel was applied on the sides of the tumor to assure better contact between electrodes and the tumor resulting in only minimal reduction of the width where the field was delivered [29,30]. After injection tumors increased in volume, however the swelling rarely exceeded 6 mm, and the tumors could still be effectively put between the electrodes.

### Irradiation of tumors

A Darpac 2000 X-ray unit (Gulmay Medical Ltd., Shepperton, UK), operating at 220 kV, 10 mA, with 1.8-mm aluminum filtration, was used for local tumor irradiation. Tumors were irradiated at a dose rate of 2.16 Gy/min with single doses in a range from 2.5-45 Gy [31]. In order to expose the whole tumor to the radiation, it was necessarily to expose some of the healthy tissue (3 – 5 mm of skin surrounding the tumor) as well.

### Treatment protocol and treatment evaluation

Radiation dose response was determined for graded single dose tumor irradiation alone (2.5-45 Gy) or combined with intratumoral mIL-12 gene electrotransfer (2.5-30 Gy) and 7 – 10 animals per treatment group were used.

Separate experiments were performed combining gene electrotransfer with 10 Gy tumor irradiation using 9-15 animals per treatment group (Tables 1 and 2). Based on our previous study [17], gene electrotransfer was performed on day 0 for single, and days 0, 2 and 4 for multiple treatments and tumors were irradiated on day 1. The antitumor effectiveness of the therapies was followed by measurement of three perpendicular tumor diameters and calculation of tumor volume using the formula V = a*b*c*п/6 [32,33]. The tumor growth delay for each experimental group was calculated as the difference in tumor doubling times of experimental and control groups. Tumor doubling time is the number of days in which the initial tumor volume (40-50 mm^3^) doubles. Unpalpable tumors were defined as complete responses. Mice that remained tumor free for 100 days were termed cured and local tumor control was deemed to have been achieved. The tumor control dose (TCD_50_) value is the dose required to control 50% of the tumors locally (i.e. cure 50% of mice). The dose modifying factor is the relative reduction of the radiation dose at the level of TCD_50_ by combination therapy compared to irradiation only. Cured mice (tumor free for 100 days) from experiments listed in Tables 1 and 2 were challenged at day 100 with a subcutaneous injection of 5 × 10^5^ SA-1 cells into the left flank and monitored for another 100 days. Animal weight was followed as a measure of the general index of toxicity.

### Cytokine measurements

A new set of experiments was performed in order to measure the cytokine concentrations in serum and the tumors. The same treatment groups were used as before (Table 1) with 8–9 animals per treatment group. Five days after the start of the therapy, blood was collected from the intraorbital sinus of all the animals, the animals were sacrificed, and whole tumors were excised. Tumors were separated from the skin. Additional experiment was performed for treatment groups that included the application of plasmid encoding IL-12 with 6-12 animals per each time point of individual treatment group. Blood and tumor samples were collected at days 3, 5, 7, 10 and 14 after the start of therapy.

Tumor and serum samples were stored at −80°C until further analysis. Tumors were mechanically macerated in liquid nitrogen, dissolved in 0.5 ml PBS containing protease inhibitors (Complete Mini, Roche, IN, USA) and centrifuged at 3000 rpm for 10 min [29]. Supernatants were removed and stored at −80°C until analysis. Tumor and serum concentrations of cytokines mIL-12 and mIFNγ were determined using Quantikine® Mouse IL-12 p70 Immunoassay (R&D Systems, MN USA). Concentrations of cytokines were calculated as pg/ml for serum samples and pg/g of tumor tissue for tumor samples.

### Histological analysis

A new set of experiments was performed in order to evaluate the histology of tumors in the control group and after intratumoral mIL12 gene electrotransfer alone or combined with tumor irradiation. Six mice per treatment group were sacrificed 5 days after the start of the therapy and whole tumors were excised and processed as previously described [17]. Tissue sections stained with hematoxylin and eosin (H&E) were analyzed by an experienced pathologist to determine changes in tumor cell morphology, the areal of tumor necrosis and viable part of the tumor, as well as the inflammatory cell infiltration.

### Skin reaction

Acute skin reaction in the whole irradiation field around the tumor was evaluated in the period from 8 to 58 days after the irradiation in all mice used for dose response evaluation. The skin reaction was scored on a scale ranging from 0 to 5 (0 – no reaction, 1 – edema, mild erythema, 2 – edema, moderate erythema, dry skin desquamation affecting < 20% of irradiated skin; 3 – edema, severe erythema, dry skin desquamation affecting > 20% of irradiated skin, 4 – edema, severe erythema, moderate moist desquamation with ulceration affecting 20-50% of irradiated skin, 5 – edema, severe erythema, severe moist desquamation with ulceration affecting > 50% of irradiated skin) adapted from [31].

### Statistical analysis

Sigmaplot 12 software (Systat Software GmbH., San Jose, California) was used for statistical analysis. All data were tested with the Kolmogorov-Smirnov test for normality of distribution. Differences between mean values of experimental groups were tested with one-way ANOVA, followed by the Holm Sidak test for multiple comparisons. Values of p < 0.05 were considered significant.

## Results

### Antitumor effectiveness and radiation response after single mIL-12 gene electrotransfer

**Figure 1:**
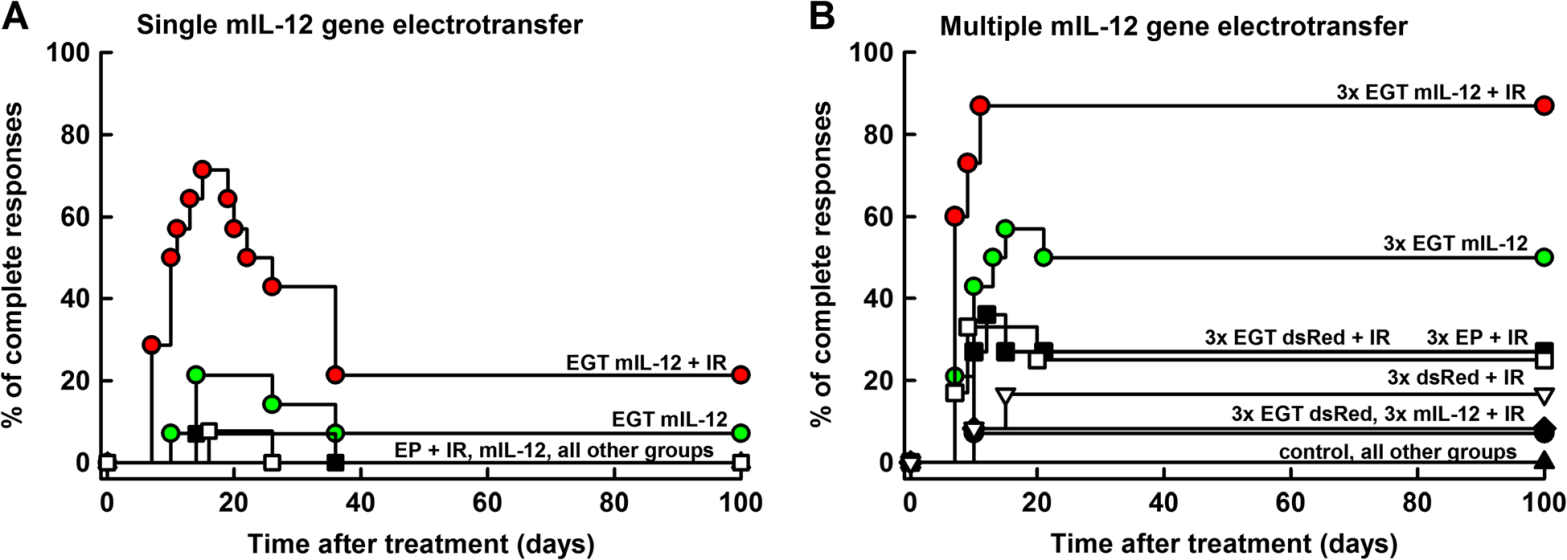
**Complete responses of SA-1 tumor bearing mice after combined modality treatment.** Single (panel **A**) and repetitive (panel **B**) intratumoral mIL-12 gene electrotransfer was used alone or combined with tumor irradiation. *Abbreviations:* mIL-12 = intratumoral injection of plasmid DNA coding for mIL-12; EGT mIL-12 = intratumoral mIL-12 gene electrotransfer; dsRed = intratumoral injection of non-therapeutic plasmid DNA; EGT dsRed = intratumoral dsRed gene electrotransfer; EP = electric pulse application on the tumors; IR = single dose radiation (10 Gy); All other groups = other therapeutic groups listed in Tables 1 and 2; 3× = triple therapy. Number of animals per treatment group is listed in Tables 1 and 2.

Single intratumoral mIL-12 gene electrotransfer in murine SA-1 sarcoma tumors resulted in 7.1% (1/14) tumor cures and when combined with tumor irradiation in 21.4% (3/14) tumor cures. In both treatment groups, a temporary and transitory increase in the complete response rate was observed 2-5 weeks after the treatment, reaching 21.4% after mIL-12 gene electrotransfer alone, and 71.4% after the combined treatment (Figure 1A). In addition, the doubling time of the remaining tumors in both treatment groups was increased significantly (p < 0.05) compared to untreated tumors (Table 1). Mice cured with the combination therapy were rechallenged with SA-1 sarcoma cells 100 days after the treatment, and one third of them (1/3) were resistant to secondary challenge. The mouse that was cured with single mIL-12 gene electrotransfer alone was not resistant to secondary challenge.

Tumor irradiation alone or combined with electric pulse application did not result in tumor cures, however tumor doubling time was significantly increased after tumor irradiation combined with electric pulse application, compared to the untreated tumors. In other therapeutic and control groups presented in Figure 1A and Table 1, no complete responses and no statistically significant change in tumor growth delay compared to untreated tumors were observed.

### Cytokine levels in tumors and serum

**Figure 2:**
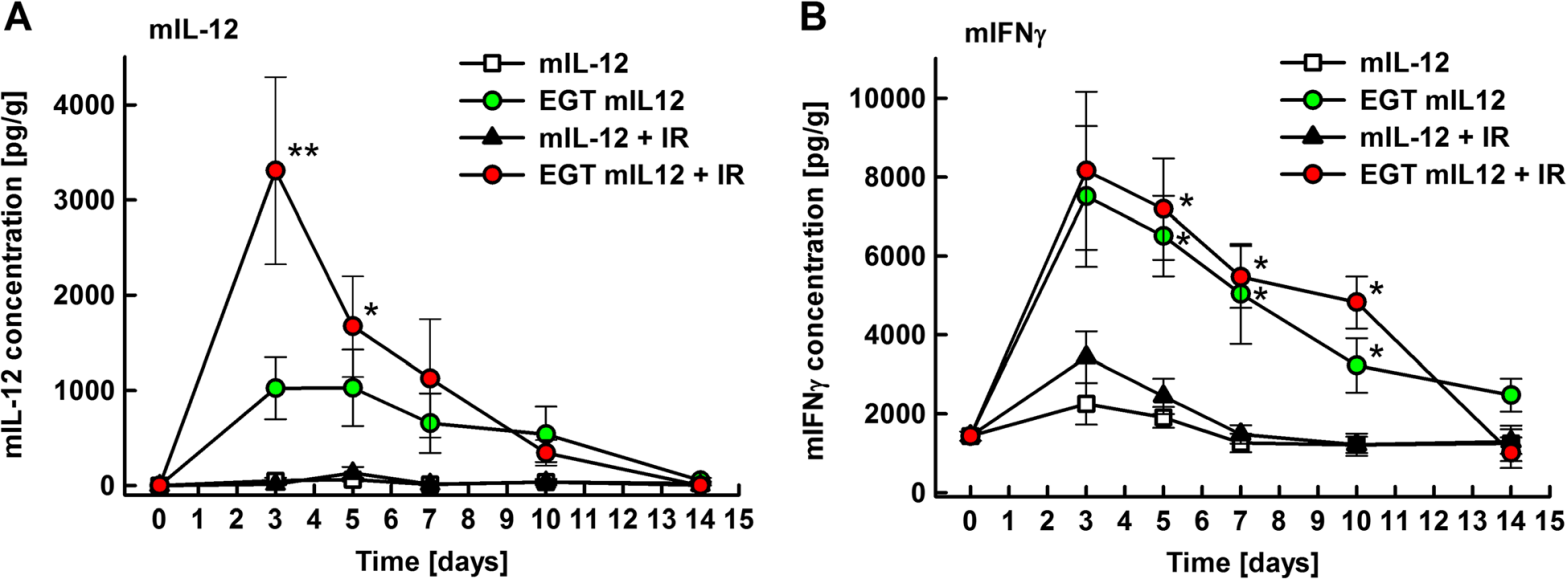
**Intratumoral concentration of cytokines mIL-12 (panel A) and mIFNγ (panel B).** Panel **A**: * - Statistically significant difference (p < 0.05) compared to groups mIL-12, mIL-12 + IR. ** - Statistically significant difference (p < 0.05) compared to groups mIL-12, mIL-12 + IR, EGT mIL-12; Panel **B**: * - Statistically significant difference (p < 0.05) compared to groups mIL-12, mIL-12 + IR. Number of animals per group was 12 (days 0, 3, 7, 10), 18 (day 5), 6 (day 14)

The concentration of mIL-12 and mIFNγ in tumors was determined in all therapeutic and control groups at day 5 after the start of therapy (Table 3). Significantly elevated concentrations of mIL-12 and mIFNγ (p < 0.05) were detected in the tumors treated with mIL-12 gene electrotransfer alone (1027 ± 400 pg/g and 6509 ± 1018 pg/g, respectively) and combined with tumor irradiation (1671 ± 529 pg/g and 7191 ± 1283 pg/g, respectively). Cytokine levels in all other groups (listed in Table 1) did not exceed 330 pg/g for mIL-12 and 2820 pg/g for mIFNγ. In the serum of the treated animals, no elevated concentrations of mIL-12 were detected in any of the therapeutic groups compared to untreated animals, and mIFNγ concentrations were below the level of detection (data not shown). In order to further elaborate the cytokine dynamics, serum and tumor concentrations of mIL-12 and mIFNγ were measured at different time points after mIL-12 gene electrotransfer alone or combined with tumor irradiation (Figure 2). The intratumoral concentrations peaked for mIL-12 and mIFNγ at day 3 post-treatment and within 14 days decreased to pre-treatment levels (Figure 2). Higher mIL-12 levels (p < 0.05; Figure 2A) were detected in the combined modality treatment group than in the mIL-12 gene electrotransfer group; however, no difference in peak mIFNγ levels was observed (Figure 2B). Again, no elevated concentrations of mIL-12 were detected in the serum of the treated animals in any of the therapeutic groups, even in the first few days after the start of therapy, and mIFNγ concentrations were below the level of detection (data not shown).

**Table 3:** Concentration of cytokines in tumors 5 days after the combined modality treatment

**Therapeutic group**	**mIL-12**	**mIFNγ**
Control groups	< 330 pg/g	< 2820 pg/g
EGT mIL-12	1027 ± 400 pg/g *	6509 ± 1018 pg/g *
EGT mIL-12 + IR	1671 ± 529 pg/g *	7191 ± 1283 pg/g *

Therapeutic groups: mIL-12 gene electrotransfer alone (EGT mIL-12) or combined with tumor irradiation (EGT mIL-12 + IR). Control groups are untreated control, 10 Gy single dose irradiation (IR), electrical pulse application alone (EP) or combined with irradiation (EP + IR), intratumoral injection of plasmid DNA coding for mIL-12 or dsRed alone (mIL-12, dsRed) or combined with irradiation (mIL-12 + IR, dsRed + IR), dsRed gene electrotransfer alone (EGT dsRed) or combined with irradiation (EGT dsRed + IR).

Abbreviations:

mIL-12 – intratumoral levels of mIL-12.

mIFNγ – intratumoral levels of mIFNγ.

* Statistically significant difference compared to untreated control (p < 0.05).

Number of animals in control groups was 8 – 9. Number of animals in groups EGT mIL-12 and EGT mIL-12 + IR was pooled from two experiments and was 17–18.

### Histological analysis

**Figure 3:**
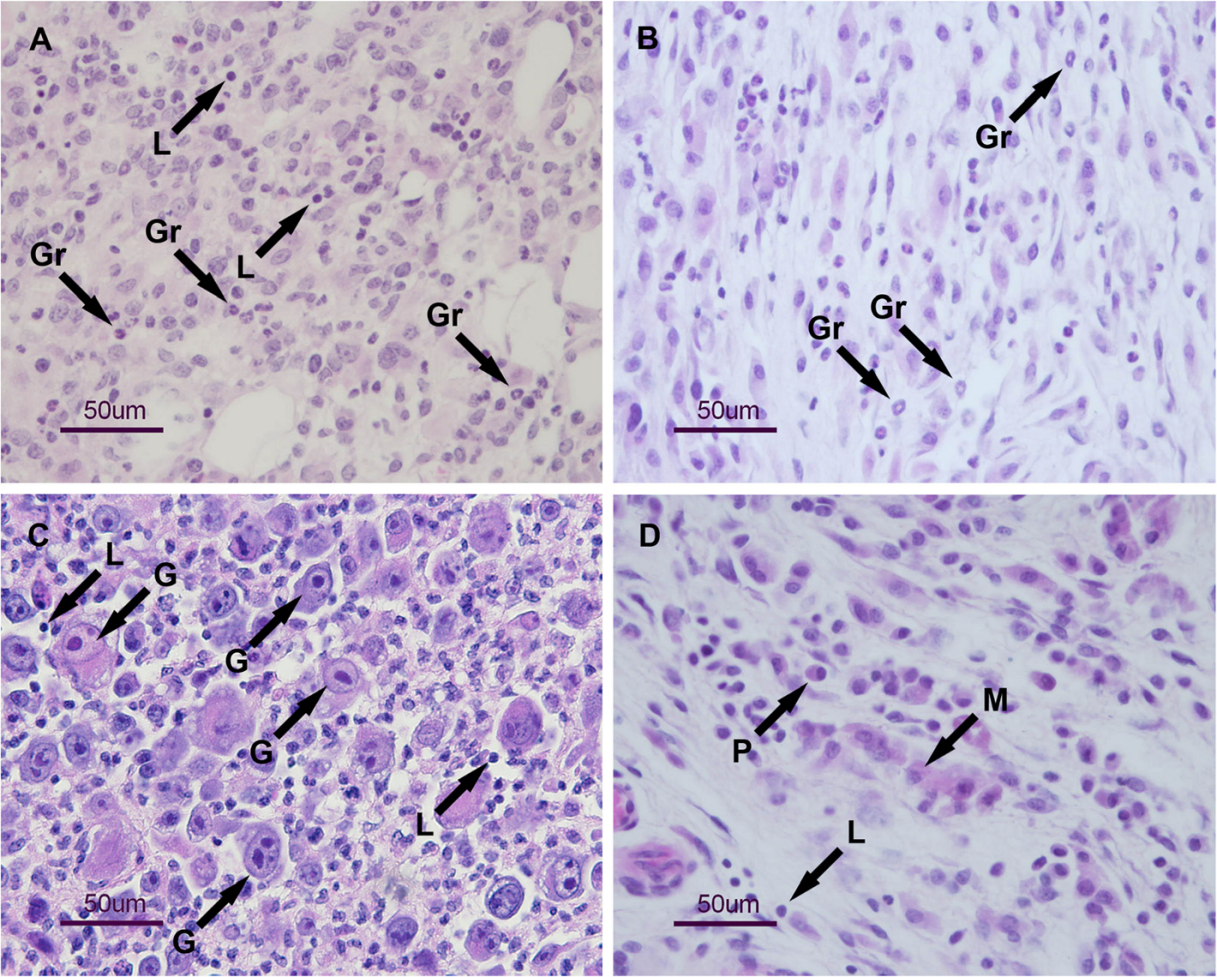
**Histology of SA-1 tumors.** Histology was evaluated at day 5 after intratumoral mIL-12 gene electrotransfer alone (**A** and **B**) or combined with irradiation (**C** and **D**). The arrows show infiltrating immune cells (L – lymphocytes, Gr – granulocytes, M – macrophages, P – plasma cells) and giant cells in mitotic arrest (G).

Variable tumor areas were seen on histological specimens at day 5 after intratumoral mIL-12 gene electrotransfer. The viable tumor area was approximately 50% of the tumor, whereas it was more than 80% in the control group. Cells with nuclear polymorphism were seen in this part of the tumor, as well as some apoptotic cells. Connective tissue ingrowing from the periphery of the tumor was observed and several inflammatory cells were found there beside fibroblasts. Lymphocytes and plasma cells predominated among inflammatory cells, although some polymorphonuclear granulocytes were also present (Figure 3A). Some infiltration of inflammatory mononuclear cells was also found between tumor cells (Figure 3B).

After mIL-12 gene electrotransfer combined with irradiation, tumors were overall smaller than tumors treated with mIL-12 gene electrotransfer but the viable tumor area was almost 90% of the tumor. However, in contrast to tumors that were treated with mIL-12 gene electrotransfer only, the cellularity of tumor cells was lower in viable areas and many apoptotic and giant cells (mitotic arrest) were seen (Figure 3C). Infiltration of inflammatory cells was also present and consisted mostly of macrophages, lymphocytes, plasma cells and individual granulocytes (Figure 3D).

### Therapeutic index

**Figure 4:**
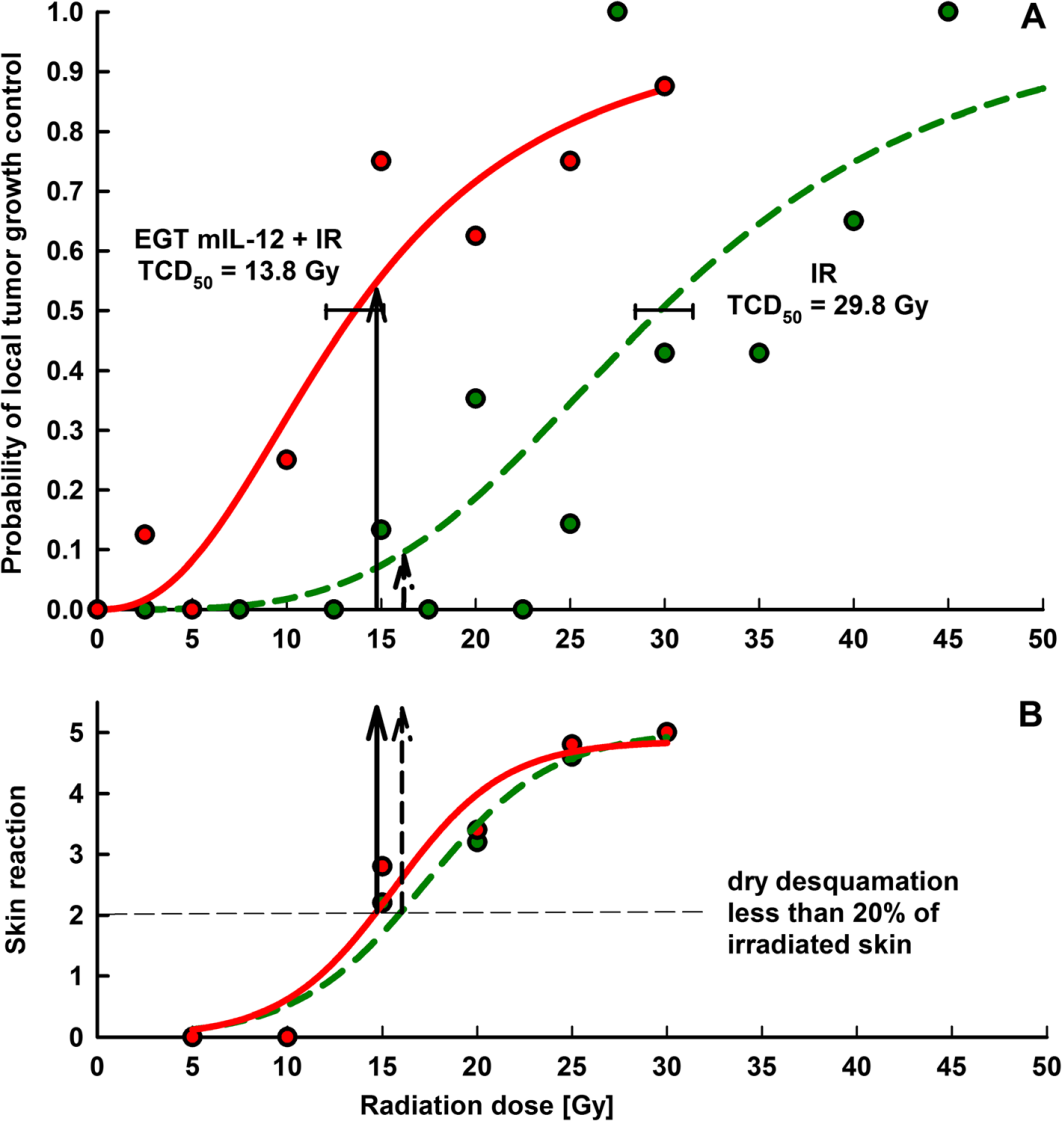
**Therapeutic index.** Radiation dose response curves for local tumor control of SA-1 sarcoma tumors (panel **A**) and skin reaction (panel **B**). Arrows indicate response on dry skin desquamation less than 20% (panel **A**), and radiation doses with which this skin reaction occurs (panel **B**). In panel A, 7–10 mice per treatment group were evaluated at each dose point. In panel **B**, 7–10 mice per treatment group were evaluated at each dose and the average of five highest skin reaction scores for each dose used was plotted.

The benefit of the combined therapy was determined by TCD_50_ assay and related to skin reaction in the tumor irradiation field. The TCD_50_ decreased from 29.8 Gy in irradiated tumors to 13.8 Gy in tumors that were additionally treated by single intratumoral mIL-12 gene electrotransfer (Figure 4A). The dose-modifying factor of a single mIL-12 gene electrotransfer in murine SA-1 sarcoma was therefore 2.16. At the same level of skin reaction, dry skin desquamation of less than 20% of irradiated skin (score 2), a 44% higher probability of local tumor control was achieved in combined therapy than with irradiation alone (Figure 4B).

Animal weight did not significantly change after intratumoral mIL-12 gene electrotransfer alone, or combined with tumor irradiation, throughout the observation time.

### Increase in radiation response after multiple mIL-12 gene electrotransfer

Multiple intratumoral mIL-12 gene electrotransfer resulted in 50% tumor cures, with a temporary increase of the complete response percentage up to 57.1% at day 15 (Figure 1B). The combined modality treatment with tumor irradiation resulted in 86.7% tumor cures. The doubling time of the remaining tumors in both treatment groups was increased significantly (p < 0.05) compared to untreated tumors (Table 2). Cured mice receiving multiple mIL-12 gene electrotransfer alone (7/14) or combined with tumor irradiation (13/15) were resistant to secondary challenge with SA-1 sarcoma cells (Table 2).

As expected, some of the treatments used as pertinent controls (Figure 1B, Table 2): multiple electrotransfer of control plasmid, combination of multiple electric pulse application and irradiation, as well as multiple injections or electrotransfer of control plasmid combined with irradiation of tumors, also resulted in tumor cures, but their antitumor and radiosensitizing effect was less pronounced [34,35]. Furthermore, mice cured with these treatment modalities were also resistant to the secondary challenge with SA-1 sarcoma cells (Table 2).

## Discussion

Our study demonstrates that single intratumoral mIL-12 gene electrotransfer resulted in increased intratumoral but not serum mIL-12 and mIFNγ concentrations, and induced pronounced antitumor and radiosensitizing effects in murine SA-1 sarcoma. The combined treatment resulted in a highly increased complete response rate of tumors and a radiation dose-modifying factor of 2.16. At the same level of skin reaction, dry desquamation, a 44% higher probability of local tumor control was observed in combined therapy than with irradiation alone. Multiple mIL-12 gene electrotransfer had even more pronounced antitumor and radiosensitizing effects.

Several studies have demonstrated that IL-12 is an effective radiosensitizer; on melanoma, Lewis lung carcinoma, mammary carcinoma, squamous cell carcinoma, as well as on fibrosarcoma, prostate and hepatic cancer [8,9,11-18]. The potentiation in most studies was additive or supra-additive, and in several cases also resulted in a high level of complete responses [8,9,11,17]. In addition to the use of recombinant IL-12, studies have predominantly been done with IL-12 gene therapy using viral vectors, which were injected intratumorally [8,9,11-13]. Intratumoral IL-12 gene transfer was also performed in our study, but using naked plasmid DNA and electric pulse application as a delivery system. Single mIL-12 gene transfer before tumor irradiation resulted in 21.4% tumor cures, while an additional two transfections after irradiation contributed to overall response, resulting in 86.7% tumor cures. The high level of complete responses obtained is comparable to the study in murine fibrosarcoma AG104A, in which single intratumoral adenoviral mIL-12 gene transfer increased the tumor cure rate of fractionated radiation (5 fractions of 5 Gy) from 26% to 59% [9].

Despite the proven efficiency of IL-12 mediated antitumor and radiosensitizing effect, the underlying mechanisms of radiosensitization remain to be elucidated, due to the complex nature of IL-12 signaling and the differences in effects observed in different tumor models [20,36]. In previous IL-12 radiosensitization studies, two main mechanisms have been hypothesized as being responsible for the effect. The first was improved immune response to tumor cells based on a significant reduction of the IL-12 mediated antitumor effect in a T-cell or NK-cell depleted environment [8] and the development of antitumor resistance after the combined treatment modality [9]. A possible explanation is that radiation induced apoptosis [12] might stimulate the immune response by creating more available tumor antigen in the tumor microenvironment [37]. The second mechanism, synergistic interaction of the IL-12 anti-angiogenic effects and radiation was hypothesized as being the main mechanism in fibrosarcoma tumors, based on decreased vascularity observed with anti-CD31 staining of tumor sections [9]; the effect, however, was not so prominent in B-16 melanoma tumors [8].

In our study, we observed increased concentration of mIL-12 in the tumors after the combined modality treatment with tumor irradiation. Increased production of IL-12 in the antigen presenting cells has been demonstrated in whole body irradiated mice [38] however in the present study tumor irradiation alone did not result in increased levels of cytokines. Radiation following gene electrotransfer might increase the production of mIL-12 in the transfected cells (tumor and stromal) however it might also influence transfection efficiency in a negative way by damaging or killing a certain percentage of the transfected cells. However radiation induced cell death does not necessarily occur immediately and it may take several days for the cells to die. Results of this and other studies [17], applying gene transfection prior to radiation, show that the combined modality treatment in this sequence results in a pronounced radiosensitizing effect. In the case of immune-modulators the radiosensitization can be temporally separated and still provides radiosensitization through potentiation of radiation elicited cell damage and by stimulation of the immune system. The increase in mIL-12 production after the combined treatment modality could also be due to the stimulation of IL-12 production in the inflammatory cells observed in the histological sections of the tumors. After combined treatment modality presence of several kinds of immune cells was observed in tumors that were not present in the control group indicating on infiltration. In our study the type of lymphocytes was not identified however previous publications using immunohistochemistry have demonstrated presence of both CD^4+^ and CD^8+^ T cells in the tumors after intratumoral mIL-12 gene electrotransfer [39]. Infiltration with CD^8+^ T cells is necessary for complete tumor eradication and CD^4+^ T cells help in achieving adaptive immune response [20,36]. Since in our experiments tumors were successfully eradicated, and development of anti-tumor resistance was observed, we can presume that both CD^4+^ and CD^8+^ cells were present. Furthermore, immune cells observed in the tumors (Figure 3) are known to produce IFNγ upon stimulation with IL-12, which could explain the elevated levels of IFNγ, demonstrated after mIL-12 gene electrotransfer (Figure 2B).

It has been shown that the antitumor effectiveness of the combined treatment with IL-12 and tumor irradiation depends on the presence of T and NK cells [8], both of which are stimulated by IL-12. Most of the IL-12 antitumor effects are mediated by the release of IFNγ, although no significant elevation of intratumoral mIFNγ was observed after combined modality treatment, compared to gene electrotransfer alone, indicating a possible saturation effect. The better response of the combined treatment modality might be explained by the antitumor and anti-angiogenic mechanisms of IL-12, which are independent of IFNγ [40].

Development of antitumor resistance after the combined treatment modality was also observed in our study [9]. One of the three tumors cured with single mIL-12 gene electrotransfer combined with tumor irradiation was resistant to secondary challenge which indicates on long-term immunity. All the mice cured by multiple mIL-12 gene electrotransfer alone or combined with radiation were also resistant to secondary challenge, however the same phenomena was observed with mice treated with treatments used as pertinent controls (multiple therapies with electric pulse application or plasmid coding for dsRed combined with tumor irradiation) that resulted in tumors cures, due to their immunogenicity. This indicates that multiple mIL-12 gene electrotransfer alone or combined with irradiation and also treatments used as pertinent controls might have either increased the immunogenicity of SA-1 sarcoma [28,41] (increased tumor antigen availability after radiation induced apoptosis) or stimulated the immune cells (control plasmid coding for immunogenic protein) [34,42,43] leading to a better tumor immunosurveillance and prevention of tumor outgrowth after secondary challenge. Thus, in contrast to the effects of IL-12 on tumor cures its contribution to the long term immunity could not be evaluated.

Radiosensitization of sarcomas is relevant in tumors that exceed the size for therapeutic effectiveness of surgery or radiotherapy as a sole treatment [25-27]. Intratumoral gene therapy with IL-12 may in such cases, as demonstrated in this study, contribute to the antitumor effectiveness of radiotherapy for effective local tumor control. Importantly, we demonstrated that skin reaction in the irradiation field was not increased by combined treatment that resulted in a dose-modifying factor of 2.16. Intratumoral mIL-12 gene electrotransfer has already been shown to be effective in sarcomas; 2.5 times higher mIL-12 plasmid doses (50 μg) resulted in up to 100% of local tumor control of SA-1 sarcoma, and also to have a systemic effect on distant tumors, as demonstrated by prolonged tumor growth delay of untreated tumors [28]. Another approach tested for the treatment of sarcomas is multiple intramuscular IL-12 electrotransfer, in which sustained release of IL-12 over weeks resulted in 30% of tumor cures and an almost 80% reduction of induced lung metastases [17]. Multiple intramuscular mIL-12 gene electrotransfer resulted in a radiosensitizing effect, resulting in 44% tumor cures compared to 28% of tumor cures after mIL-12 gene electrotransfer only [17]. The results of the present study therefore indicate that intratumoral mIl-12 gene electrotransfer is superior to intramuscular for local tumor control, since multiple intratumoral mIL-12 gene electrotransfer combined with irradiation resulted in 86.7% tumor cures, while the dose modifying factor of single intratumoral mIL-12 gene electrotransfer combined with irradiation was 2.16. The result is also superior to the dose modifying factor that we obtained in our previous studies on an LPB sarcoma tumor model [44,45], in which irradiation was combined with increased chemotherapeutic drug content in the tumors due to electrotransfer (electrochemotherapy) [46]. In these studies the dose modifying factor for electrochemotherapy with cisplatin was 1.6 [44] and for electrochemotherapy with bleomycin 1.9 [45]. This demonstrates that stimulation of the immune system results in more pronounced radiosensitization, with broader clinical applicability targeting local, but also systemic disease. Our study provides a proof of concept regarding the therapeutic efficiency of intratumoral mIL-12 gene electrotransfer combined with single dose tumor irradiation. However studies using fractionated radiation regime will be necessary in order to bring this treatment closer to the clinical setting.

## Conclusions

Single or multiple intratumoral mIL-12 gene electrotransfer results in increased intratumoral mIL-12 and mIFNγ cytokine levels, and may provide an efficient treatment modality for soft tissue sarcoma as a single or adjuvant therapy to tumor irradiation. Combined treatment resulted in a highly increased complete response rate of murine SA-1 sarcoma, with no significant effect on irradiation induced damage to normal tissue in the irradiation field. Furthermore, repetitive gene electrotransfer compared to single one, further potentiated antitumor and radiosensitizing effects as well as increased the resistance to secondary challenge.

## Abbreviations

TNF-α: Tumor necrosis factor alpha; IL-2: Interleukin 2; IL-12: Interleukin 12; mIL-12: Murine interleukin-12; IFNα: Interferon alpha; IFNß: Interferon beta; IFNγ: Interferon gamma; mIFNγ: Murine interferon gamma; SA-1: Murine fibrosarcoma, LPB, Murine sarcoma; AG104A: Murine fibrosarcoma; B16: Murine melanoma; PBS: Phosphate buffered saline; dsRed: Red fluorescent protein; pORF mIL-12: Plasmid coding for mIL-12; pORF dsRed: Plasmid coding for dsRed; H&E stain: Hematoxylin and eosin stain; T cell: T lymphocyte; NK cell: Natural killer cell; TCD_50_: Tumor control dose, DT, Tumor doubling time; GD: Tumor growth delay; EP: Electric pulse application; EGT: Gene electrotransfer, IR, Tumor irradiation.

## Competing interests

The authors declare that they have no competing interests.

## Authors’ contributions

AS carried out the *in vivo* studies, performed the data analysis and drafted the manuscript. SK and TD participated in the *in vivo* studies. AC carried out the histological analysis. MC, GS conceived the study, participated in the design and coordination and helped to complete the manuscript. All authors read and approved the final manuscript.

## Pre-publication history

The pre-publication history for this paper can be accessed here:

http://www.biomedcentral.com/1471-2407/13/38/prepub
